# Hybrid Organic–Inorganic Blast Furnace Slag Binders Activated with Alkali Acetates

**DOI:** 10.1021/acsomega.4c04857

**Published:** 2024-08-09

**Authors:** Yuyan Huang, Alastair T. M. Marsh, Zengliang Yue, Sreejith Krishnan, Samuel Adu-Amankwah, Susan A. Bernal

**Affiliations:** † School of Civil Engineering, University of Leeds, Leeds LS2 9JT, United Kingdom; ‡ Department of Civil and Infrastructure Engineering, Indian Institute of Technology Jodhpur, Jodhpur 342011, India; § School of Engineering and Applied Science, Aston University, Birmingham B4 7ET, United Kingdom

## Abstract

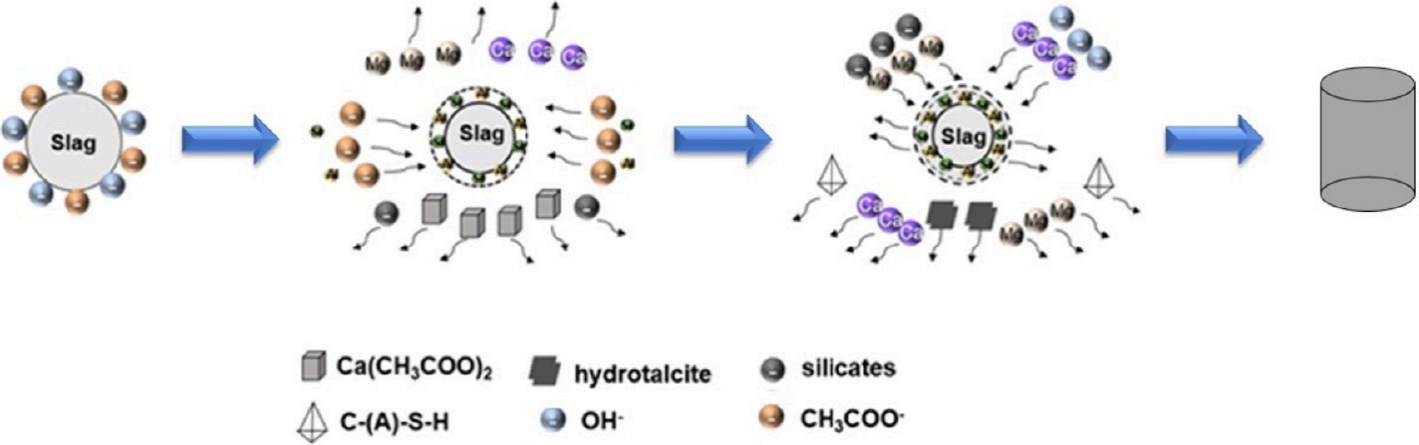

Hybrid organic–inorganic binders based on blast furnace slag were produced using sodium (NaAc) or potassium (KAc) acetate as the sole activator, and their properties were compared with those of sodium- or potassium hydroxide-activated slag pastes. The acetate-activated binders showed significantly lower cumulative heat release and extended setting time (∼230 h) than the hydroxide-activated binders. The main reaction products forming in all binders were calcium aluminosilicate hydrate-type gels and a hydrotalcite-like phase, independently of the activator type used. Compressive strengths of the acetate-activated pastes (∼40 MPa at 180 days) were lower than those of the hydroxide-activated binders (∼80 MPa at 180 days). However, the acetate-based binders exhibited superior impermeability and reduced wettability at 28 days, likely due to hydrophobic acetate groups. It is hypothesized that acetates dissociate in water, forming calcium acetate and alkali silicates via a reaction with species dissolving from the slag. This study demonstrates alkali acetates are effective activators for creating hybrid slag-based binders with reduced permeability.

## 1 Introduction

The production of alkali-activated slag cements using sodium hydroxide (abbreviated as NaOH) or sodium silicates (abbreviated as Na_2_SiO_3_) (as well as potassium equivalents) as alkaline activators has been extensively investigated due to their potential for reducing the carbon footprint compared to conventional Portland cements, when used to produce different construction products (e.g., mortars, concrete).^1,2^ However, the high alkalinity of such activators is perceived as one of the key limitations for their widespread use.^3^ To address this issue, researchers have explored different activating routes including the use of near neutral salts such as sodium carbonate (abbreviated as Na_2_CO_3_)^4,5^ or sodium sulfate (abbreviated as Na_2_SO_4_)^6^ with demonstrated potential for enhancing the durability of materials produced with them, although their effectiveness can vary depending on slag composition. Sodium aluminate (abbreviated as NaAlO_2_) has also been used as a potential activator; it is known for accelerating the setting time and early strength development,^7^ but its high cost and sensitivity to environmental conditions limit its widespread use. The use of calcium oxide (abbreviated as CaO)^8^ and magnesium oxide (abbreviated as MgO)^9,10^ has also been investigated, as the formation of calcium or magnesium bearing phases can be promoted, contributing to the mechanical strength development of slag-based cements. However, their high reactivity can pose challenges in terms of handling and workability of the pastes produced with them.^11^ This highlights the urgent need of developing studies exploring alternative routes of activation to achieve a desirable performance of end products, while finding practical solutions that can be scaled up in future construction practices.

Over the past decades, there has been a growing interest in developing hybrid organic–inorganic alkali-activated materials with the aim of enhancing reactivity and/or mechanical properties of these binders. The term “organic” is used here to broadly refer to carbon-based substances (excluding simple carbonates). These hybrid organic–inorganic materials comprise a conventional alkali-activated binder, an aluminosilicate source plus an alkali activator (often sodium silicate), and minor additions of organic substances. There are two main rationales for designing hybrid organic–inorganic alkali-activated binders: to enhance strength and toughness by filling pores with an organic phase^12^ or to enhance hydrophobicity by a combination of altering pore structure and/or the surface chemistry.^13^ The majority of studies in this area have centered in evaluating uncalcined clay-,^14,15^ metakaolin-,^13,16^ or fly ash-based^17,18^ geopolymers, and some of the organic substances evaluated include epoxy resins,^16^ polyurethane waste,^19^ melamine,^20^ and polyethylene glycol,^21^ among others.

Very limited studies on hybrid organic–inorganic alkali-activated slag cements have been conducted. Xing et al.^22^ added 0.5% to 5% of polyacrylamides (CPAM), poly(vinyl alcohol) (PVA), or poly(acrylic acid) (PAA) to sodium silicate-activated slag binders and identified an increase in flexural strength particularly when adding PVA. Chen et al.^23^ evaluated the effect in the microstructure and performance of alkali-activated slag cements of adding small quantities (0.4–1% per gram of slag) of poly(ethylene glycol) (PEG), polyacrylamide (PAM), and sodium polyacrylate (PAAS) to sodium silicate activating solutions. In this study, a reduction in the porosity and an increase in the toughness of these materials were identified. Ramaswamy et al.^24^ evaluated the effect of different ligands (e.g., triethanolamine (TEA), triisopropanolamine (TIPA), trisodium nitrilotriacetate (NTA), tetra potassium pyrophosphate (TKPP), and 2,3-dihydroxynapthalene (DHNP)) on sodium carbonate-activated slag cements. Results revealed that depending on the type of ligand added, the pH of the pore solution increases, accelerating the dissolution of Si and other metal ions from the slag via complexation reactions, accelerating the reaction kinetics and therefore the compressive strength development of these cements.

Metal acetates are organic ionic salts made up of two components: a metal cation and an acetate anion. They have been used by the construction sector as deicing and anti-icing agents, mainly for use on airfield pavements and bridge decks.^25^ Their advantage over conventional deicing salts is that they do not contain chloride anions and hence reduce the risk of chloride-induced corrosion of steel reinforcement.^26^ Sodium acetate,^25^ potassium acetate,^26^ magnesium acetate,^27^ calcium acetate,^27^ and calcium magnesium acetate^26^ have all been investigated or used in practice as deicers. However, the potential of metal acetates (especially potassium acetate) to exacerbate the alkali–silica reaction in concrete has been a subject of ongoing research.^28^ Potassium acetate has been shown to increase the OH^–^ concentration in cement pore solution through its reaction with Ca(OH)_2_ to form Ca acetate and hence increase the solubility of Ca(OH)_2_.^29^ Although there are several studies investigating the use of alkali metal acetates in concrete, mainly to enhance impermeability,^30^ the use of alkali metal acetates as the only source of alkalis to manufacture alkali-activated slag cements has not yet been explored. Alkali metal acetate solutions can provide sufficient alkalinity for the precursors to dissolve and alkali cations for charge-balancing reaction phases forming in the binder. Such actions fulfill the requirements of an alkaline activator.^31^ Therefore, it is hypothesized that metal acetates could form suitable alkaline activating solutions when dissolved in water.

Thus, this study investigated the feasibility of producing hybrid organic–inorganic blast furnace slag-based binders using sodium acetate (abbreviated as NaAc) or potassium acetate (abbreviated as KAc) solutions as sole activators. Conventional sodium hydroxide- or potassium hydroxide-activated slag cements were also produced as reference systems to determine the effectiveness of the metal acetate solutions. The reaction kinetics and phase assemblage evolution of the binders produced were evaluated by applying multiple analytical characterization techniques. Compressive strength, porosity, and water contact angle (wettability) of the cements produced were also determined.

## 2 Materials and Methodology

### 2.1 Materials

A commercial granulated blast furnace slag (GBFS) was used as the precursor in this study, with an oxide composition (Table 1) determined by X-ray fluorescence (XRF) spectroscopy (Rigaku ZSX Primus II) using the fused bead preparation method. The blast furnace slag had a specific surface area of 1.25 m^2^/g, determined by BET nitrogen sorption analysis (Micromeritics Tristar), and its *d*_50_ was 10.75 μm, measured by laser diffraction (Malvern Mastersizer 2000) in a dispersant of isopropanol.

**Table 1:** Oxide Composition (wt %) of the GGBFS Measured by XRF^a^

chemical composition									
SiO_2_	Al_2_O_3_	CaO	MgO	K_2_O	Na_2_O	Fe_2_O_3_	SO_3_	TiO_2_	LOI
36.30	9.70	43.85	6.28	0.3	0.26	0.37	1.48	0.59	0.87

a LOI is loss on ignition at 900 °C.

Sodium hydroxide (NaOH, Honeywell, 98%) as well as potassium hydroxide (KOH, Honeywell, 98%) were employed as benchmark activators. Commercial sodium acetate, abbreviated as NaAc (anhydrous, Alfa Aesar, 99%), and potassium acetate, abbreviated as KAc (anhydrous, Alfa Aesar, 99%), were used. Metal acetates are known to be highly hygroscopic.^32^ To ensure the dehydration of the metal acetate prior to being used, the acetate was heated at 350 °C in air for 7 min. This temperature is deemed sufficiently high to achieve dehydration of the hydrate phases but not high enough to incur thermal decomposition. The treated acetates are referred to as NaAc or KAc throughout the manuscript. The quantity of acetates used to produce the activating solutions was determined so that the equal number of moles of alkalis (i.e., Na^+^, K^+^) were supplied to the system by the acetate or hydroxide activating solutions. The molality and composition of the activating solutions are listed in Table 2.

**Table 2:** Composition and Molality of the Activating Solutions

alkali source	mass of solid activator (g)	mass of distilled water (g)	molality (moles of alkali source/kg of water)
NaOH	5.16	31.55	4.09
KOH	7.22	32.17	4.01
NaAc	10.58	33.17	3.89
KAc	8.34	32.50	2.62

### 2.2 Sample Preparation and Mix Design of the Evaluated Binders

The slag-based pastes were prepared with a constant water to solid (slag + solid activator) ratio of 0.3 and an activator amount of 4 wt % M_2_O (where M = Na or K) with respect to the mass of the slag, in line with ref (33). The activating solutions were prepared by dissolving the solid activator in distilled water by using a stirrer plate to ensure complete dissolution of all solids. The dissolution of NaOH or KOH pellets in distilled water causes an exothermic reaction. It is essential for the alkaline solutions to cool to room temperature to maintain consistent reactivity in subsequent processes. Therefore, the solutions were prepared at least 2 h prior to their use. Table 3 reports the mix design of the evaluated binders. The pastes were produced by mixing slag and the activating solution in a high-shear mixer for 3 min at low speed (139 rpm), followed by 2 min at high speed (591 rpm). Then, pastes were cast in 15 mL centrifuge tubes, sealed, and placed in a water bath at 20 °C for up to 180 days.

**Table 3:** Mix Proportions of Produced Slag-Based Binders

activator	slag (g)	M_2_O (wt %)	activator (g)	water/solid ratio	water (g)
NaOH	100	4	5.16	0.3	31.55
NaAc cast	100	4	10.58	0.3	33.17
KOH	100	4	7.22	0.3	32.17
KAc cast	100	4	8.34	0.3	32.50

### 2.3 Characterization of Dried Acetates and Activating Solutions Produced with Them

The dried acetates were characterized by X-ray diffraction (XRD) using a Bruker D8 diffractometer to ensure the dehydration of the hydrate phases prior to producing the activating solutions. The radiation used was Cu Kα with a wavelength of 0.1541 nm (40 kV). The 2θ range evaluated was 5° to 45° with a step size of 0.033° at a rate of 3 s/step at room temperature.

The pH values of solutions created by dissolving NaAc and KAc after heat treatment into distilled water, as well as NaOH and KOH solutions, were monitored at 5 min time intervals using a pH meter (HACH HQ40D) up to 30 min to ensure all the solids were fully dissolved.

### 2.4 Fresh State Properties of Pastes

The reaction kinetics were evaluated by isothermal calorimetry by using a TAM air calorimeter. For each mix, 9 g of fresh paste was tested at 20 ± 0.02 °C for 28 days. Reference samples were composed of quartz and distilled water (9 g in total). The heat release was normalized by the total mass of the paste.

The initial and final setting times were determined using a Vicat apparatus, coupled with a 1.13 mm diameter needle, according to BS EN 196-3:2016.^34^ When nearing the initial setting time, measurements were taken every minute, similarly for the final setting time. The typical configuration of the Vicat apparatus for measuring setting time, in which the paste sample is immersed in water, is not suitable for alkali-activated pastes because the submersion in water would lead to leaching of the activator and hence affect the reaction kinetics. Instead, the paste was placed in a fog room with high relative humidity (>90%) at approximately 20 °C.

The flowability of freshly prepared workability of the alkali-activated slag (AAS) pastes was determined by a mini-slump test.^35^ The test apparatus consisted of a cylindrical container (5 cm in diameter and 10 cm in height) and a 2 × 2 cm^2^ square poly(methyl methacrylate) flat plate with markings. Approximately 70 g of freshly mixed paste was used for each measurement. For each mix design, three freshly prepared batches of pastes were evaluated. The yield stress has previously been quantitatively linked with the mini-slump test.^36^ The yield stress (*τ*_0_) of the binders assessed was calculated according to eq 1,^35^ considering the density of paste ρ, the volume of the cone Ω, and the spread diameter *R*. *g* represents the acceleration due to gravity, assumed to be 9.81 m/s^2^.
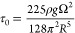
1The density of every paste mix was calculated considering the density of every component according to ref (37).

### 2.5 Phase Assemblage Evolution and Microstructural Analysis

The pastes were cured for 7, 28, 60, and 180 days in sealed containers in a water bath at 20 °C. After different curing durations, the hardened pastes were manually ground into powders until they passed through a 74 μm sieve to obtain similar fineness prior to testing.

A Bruker D8 powder X-ray diffractometer (using Cu Kα radiation, λ = 0.1541 nm) operating at 40 kV was used to collect diffraction patterns. The range evaluated was from 5° to 60° (2θ), using a step size of 0.033° (2θ) at a rate of 3 s/step. All measurements were conducted at room temperature. The HighScore software and the PDF-4+ 2023 ICDD database were utilized for crystalline phase identification.

Mid-range Fourier transform infrared (FTIR) spectra were collected using a PerkinElmer Spectrum 2 spectrometer with an ATR attachment in the range of 400–4000 cm^–1^ at a resolution of 4 cm^–1^ with a minimum of 16 scans per measurement.

The solid-state ^29^Si MAS NMR spectra of 180 day cured samples were collected in a Bruker Avance III HD spectrometer with a 400 MHz magnet (magnetic field: 9.4 T). Powder samples were packed in a zirconia rotor used in a 7 mm probe rotating at 6 kHz. Measurement conditions were a 79.5 MHz operating frequency, a 90° pulse of 5.5 μs duration, and a 40 s relaxation delay with at least 2048 scans collected for each spectrum. The reference for the ^29^Si shifts was tetramethylsilane (TMS). Conventional Q^*n*^(*N*Al) notation is employed to describe Si bonding, where *n* denotes the number of −O–Si “bridges” to the closest neighboring sites and *N* indicates the number of Al next-nearest-neighbor sites.^38^ Deconvolutions of the ^29^Si MAS NMR spectra were not performed in the study due to the relatively low pH values of the acetate-based activating solutions; therefore, congruent dissolution of the slag cannot be assumed.

Scanning electron microscopy using a backscattered electron (BSE) detector coupled with energy dispersive X-ray spectroscopy (EDS) was employed for studying the microstructural and compositional variations in the cured pastes. Sections of each cured paste specimen were impregnated with an epoxy resin. SiC paper with grit sizes of P600, P1200, and P2500 were used for initial grinding of the exposed surface, followed by a sequence of diamond pastes containing progressively smaller particle sizes (6, 3, 1, and 0.25 μm) for the polishing step. A Zeiss Evo 15 scanning electron microscope operating at a voltage of 20 kV was used for image analysis. The samples were evaluated after 180 days after being sputter coated with gold. Element maps of calcium, aluminum, magnesium, sodium or potassium, silicon, and carbon were collected.

### 2.6 Mechanical and Physical Characterization

Paste samples were cast into 25 × 25× 25 mm^3^ molds for compressive strength testing. Molds were sealed with plastic wrap and cured in a fog room (95% relative humidity) for 7, 28, 60, and 180 days. An Instron 3382 instrument was employed for the compressive strength measurements, using a loading rate of 50 N/s, in line with BS EN 196-1:2016.^34^

The pore size distribution of samples cured for 28 days was determined by mercury intrusion porosimetry (MIP) using a MicroActive AutoPore V 9600 Version 1.02 instrument. In this study, cylindrical samples with an aspect ratio of 1:1 were cut and then quartered. The small specimens were placed in a 60 mL beaker, covered with ethanol, and sealed for 5–6 h. Samples were then placed in a vacuum desiccator before testing for 1 week. A mass of 0.8–1 g was used per test using a Penetrometer 11 with a 5 cc head size. Mercury was intruded at a rate of 0.1–61 000 psi at 20 °C, and a contact angle was set at 130°.

Water contact angle was measured to determine the potential hydrophobicity or hydrophilicity of pastes cured for 28 and 180 days. Water contact angle refers to the angle that a liquid interface meets a solid surface.^39^ Disks 2 mm in thickness were cut by using an Isomet high-precision saw. The basal surfaces of each disk were polished by using SiC paper to flatten the surface and reduce potential roughness induced by cutting, which can influence the contact angle detected. After a flat surface was obtained, the samples were kept in a vacuum desiccator before testing. Images with contact angle values were automatically collected by using a KSV instrument for each water contact angle measurement. The released water drop volume was 0.10 mL per measurement.

## 3 Results and Discussions

### 3.1 Metal Acetate Activating Solutions

**Figure 1:**
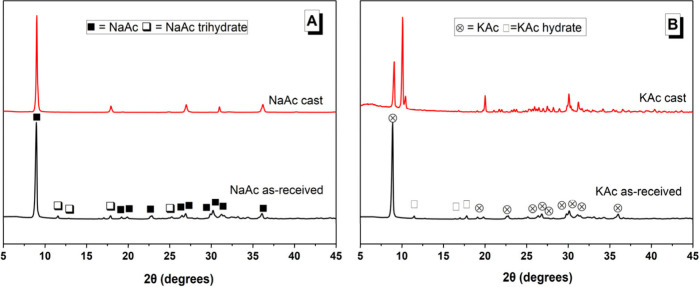
Cu Kα XRD patterns of (A) NaAc and (B) KAc. The word “cast” refers to samples after heat treatment.

The XRD patterns of NaAc and KAc, both in their as-received form and after the initial heating step to remove any hydrate phases, are shown in panels A and B of Figure 1, respectively. For both NaAc and KAc, the heat treatment was successful at dehydrating the hydrate phases present in the as-received materials, with the anhydrous phases of sodium acetate (powder diffraction file (PDF) #00-028-1029) and potassium acetate (PDF #00-046-0898) remaining.

In KAc, an additional strong reflection was observed at 10.08° (2θ); the limited number of reference patterns for potassium acetate meant this could not be identified with confidence. At least two polymorphs of potassium acetate are known to exist above room temperature;^40^ the partial retention of a higher-temperature polymorph after rapid cooling is plausible.

**Figure 2:**
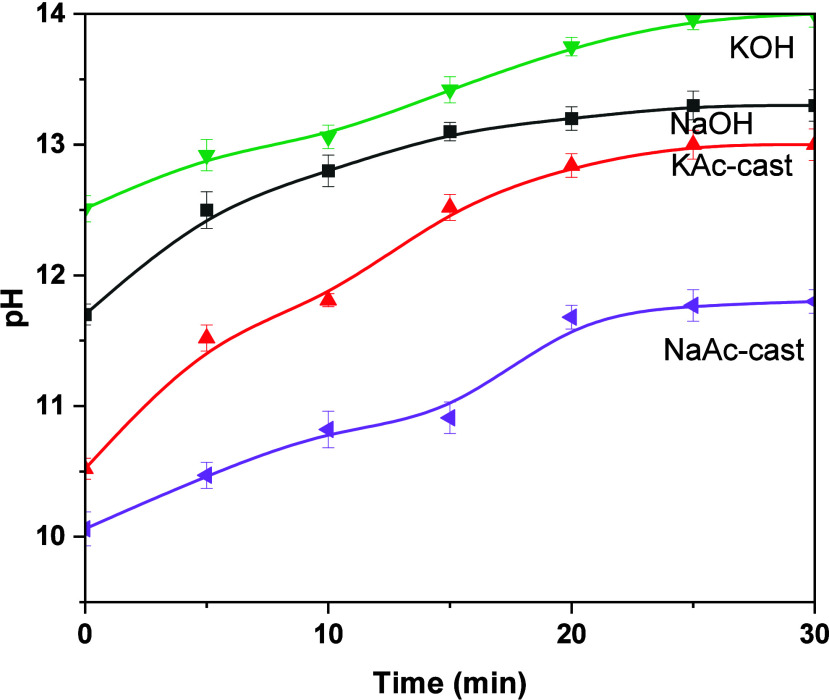
pH value of the activating solutions up to 30 min after mixing.

Activating solutions were prepared by adding the as-received hydroxide activators and dehydrated acetate activators to distilled water (Table 2). The measured pH values up to 30 min are shown in Figure 2. The pH value of all the solutions became stable 30 min after mixing, indicating that dissolution was complete. The ranking of pH values after 30 min, from strongest to weakest, was KOH (14.0), NaOH (13.3), KAc (13.0), and NaAc (11.8). The NaAc solution had the lowest pH value, despite its molality being higher than the KAc solution. This difference may arise from buffering reactions induced by the dissolution of atmospheric carbon dioxide.^41^

The alkaline pH of the acetate salts is advantageous to promote alkali activation reactions by contributing hydroxide ions to the solution, effectively elevating the alkalinity of the solution. Hydroxide ions, along with acetic acid, are generated by a series of dissociation reactions (eqs 2 and 3).^41^ This, in turn, can enhance the dissolution of silicate or aluminosilicate present in the slag, which is a fundamental step in the alkali activation reaction process.
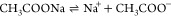
2
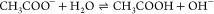
3

The pH values of the NaAc and KAc solutions were both ≤1 unit lower than their respective hydroxide activating solutions. The molality of the NaAc and KAc activating solutions was 3.89 and 2.62 mol/kg, respectively (Table 2). Considering that the solubility limits of sodium acetate or potassium acetate under standard temperature and pressure are 123.3 g/100 mL and 268.6 g/100 mL, respectively,^42^ there is potential to develop higher-concentration solutions of NaAc or KAc with molality of up to 15.03 and 27.41 mol/kg. However, to determine the effectiveness of these solutions in comparison with conventional hydroxide-based ones, the molarity concentrations or amount of alkalis (Na^+^ or K^+^) were dosed at values consistent with current practice when producing alkali-activated slag cements.

Nonetheless, it is important to note that highly alkaline solutions are not essential for producing slag-based binders due to the hydraulic nature of blast furnace slag. The activating solution accelerates the rate of the slag reaction and provides cations and anions that can influence the phase assemblage.^43^ While the lower pH of the acetate activators may make them unsuitable for pozzolanic aluminosilicate precursors (e.g., calcined clays), which require high pH for acceptable dissolution rates, they can be acceptable for hydraulic precursors (e.g., blast furnace slag).^44^

### 3.2 Reaction Kinetics and Fresh State Properties

**Figure 3:**
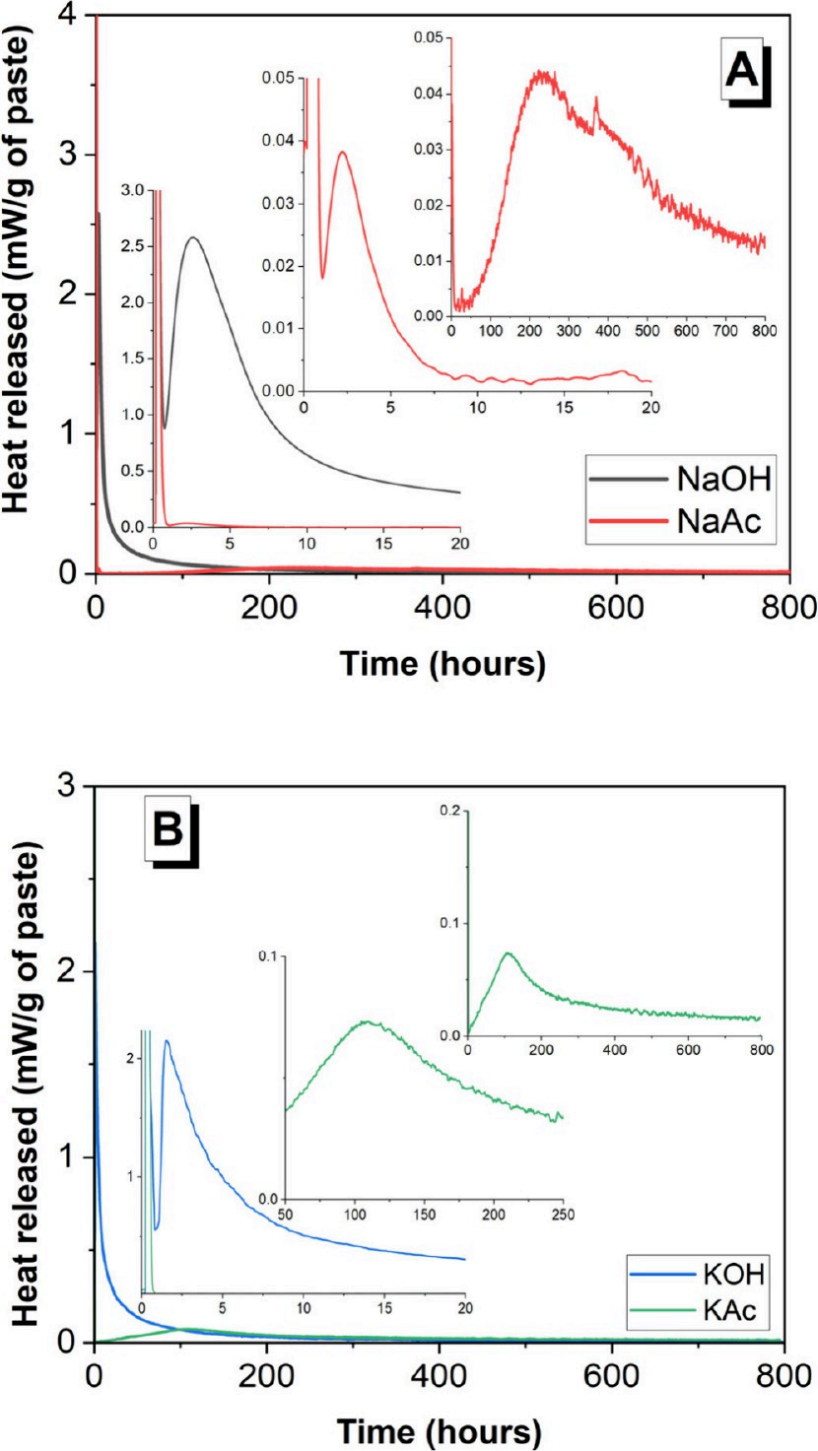
Heat release rate of AAS produced with different activators including (A) NaOH and NaAc and (B) KOH and KAc (relative to the mass of mixed paste). Time is after mixing time.

**Figure 4:**
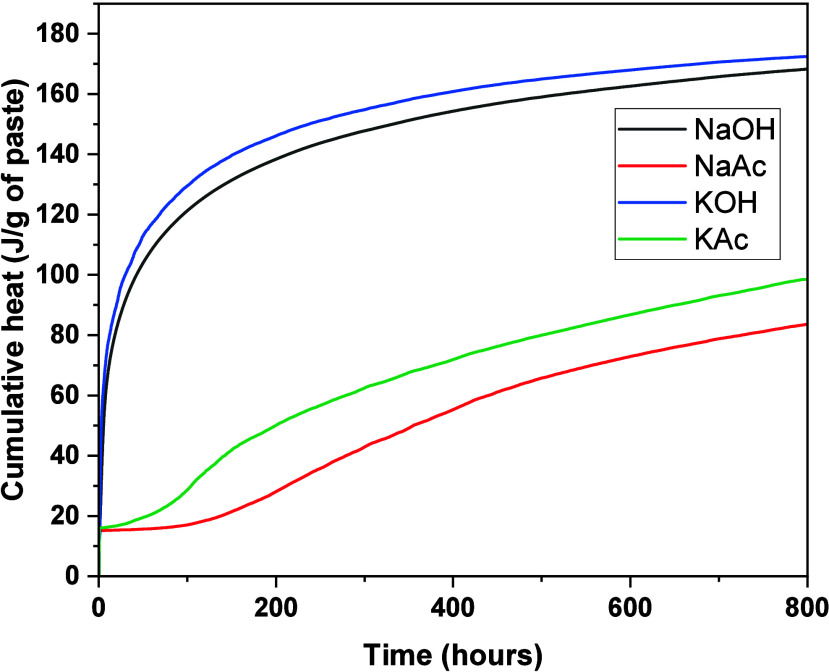
Cumulative heat of slag-based binders produced with different activators; 800 h is equivalent to 33.3 days.

An initial heat release peak (<1 h) was observed in all isothermal calorimetry curves (Figure 3), consistent with the wetting and dissolution of slag.^45^ In the hydroxide-activated pastes, the induction period was followed by an acceleration period, typically associated with the formation of reaction products, most likely aluminum-substituted calcium silicate hydrates (C-(A)-S-H).^45^ The onset of acceleration occurred at approximately 1 h in both hydroxide-activated slag pastes; the corresponding exothermic peak centered is observed at 2.5 h in the NaOH-activated slag and 2 h in the KOH-activated slag binder. Beyond the time of maximum heat flow in the acceleration period, the heat flow decreased to a low yet non-negligible value, as seen in the continuing increase in the cumulative heat curves (Figure 4). The features of the heat curves of the hydroxide-activated slag cements are consistent with those observed in previous studies.^46^

In the NaAc-activated slag binder (Figure 3A), the induction period was followed by an acceleration period with its peak center at 2.5 h. The time of maximum heat release, associated with the acceleration period, was similar to that of the NaOH-activated slag binder but 2 orders of magnitude smaller (0.038 mW/g of paste) compared to the NaOH-activated binders (2.6 mW/g of paste). A dormant period then occurred. After nearly 50 h, a second acceleration period began with a slow increase in heat flow up to a maximum of 0.044 mW/g of paste at 228 h, after which heat flow slowly decreased.

In the KAc-activated slag binder (Figure 3B), no acceleration period was observed within the first 5 h after mixing, conversely to the NaAc binder, which exhibited a modest acceleration period within the first 5 h of reaction. After a dormant period of approximately 40 h, an acceleration period began with a slow increase in heat flow up to a maximum of 0.07 mW/g of paste at 128 h, followed by a decrease in heat flow. The time at which maximum heat flow occurred in this delayed acceleration period was noticeably earlier for KAc (128 h) compared to NaAc (228 h). Nonetheless, this delayed acceleration period was a shared feature of both acetate-activated slag binders.

The cumulative heat values (Figure 4) recorded for the NaAc- or KAc-activated pastes (83.63 and 98.48 J/g of paste) were significantly smaller than those recorded for the NaOH- or KOH-based pastes (168.28 and 172.43 J/g of paste).

The lower pH of the acetate-based activators (Figure 2) may have delayed the dissolution of the slag, consequently resulting in a longer time to reach the critical concentration of ionic species in solution to form reaction products.^47^ However, given that the pH value of the KAc activating solution (13.2) was not substantially lower than the pH of the KOH activating solution (14.0), it is unlikely that a lower alkalinity alone explains the significant difference between the acetate- and hydroxide-activated slag pastes. It is possible that acetate anions are reacting with species released from the slag (i.e., Ca, Si, Al), thus delaying the onset of supersaturation and hence hindering the precipitation of reaction phase(s). These isothermal calorimetry results indicate that the sequence of solution-mediated reactions and dissolution of slag when using acetate-based activators are different from what are known for hydroxide-based activators. This is discussed in more detail in section 3.6.

The setting times of the pastes evaluated are reported in Table 4. The NaOH-activated binders had an initial setting time of ≤0.5 h, consistent with previous studies analyzing samples produced with similar activators^48^.^49^ The initial setting times for the acetate-activated samples were much longer: 3 h for the NaAc binder and 1.5 h for the KAc binder respectively.

**Table 4:** Setting Time of Activated Slag Binders Produced with Various Activators

activator type	initial setting time (h)	final setting time (h)
NaAc	3.0	230
NaOH	0.5	3
KOH	0.2	4.5
KAc	1.5	129

The final setting times for NaOH- and KOH-activated pastes were 3 and 4.5 h, respectively. The acetate-activated samples both had far longer final setting times (>100 h) than the hydroxide-activated samples. The final setting times align closely with the time of maximum heat flow in the acceleration period for each paste, as shown in Figure 3A,B. The long setting time of the acetate-activated slag pastes may restrict their use in some, but not all, applications.

**Figure 5:**
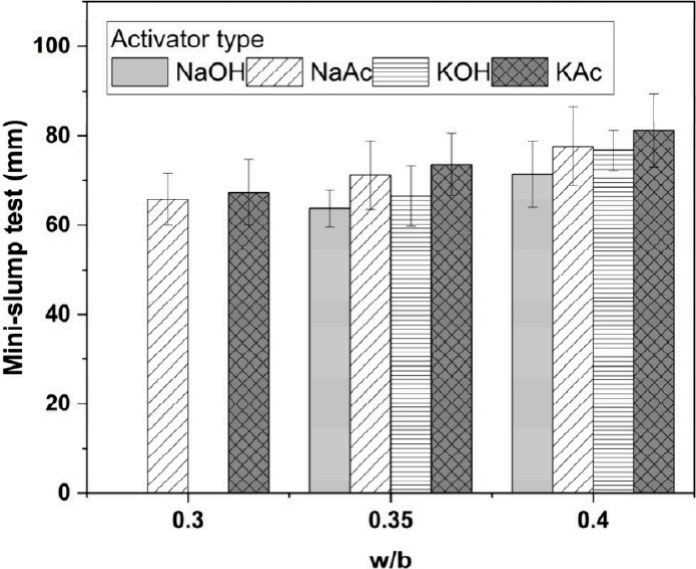
Effect of w/b ratio on mini-slump of alkali-activated slag pastes produced with different activators. Results for NaOH- or KOH-activated pastes with w/b = 0.3 are not reported, as the pastes were not fluid.

The mini-slump spread diameters of activated slag pastes as a function of water to binder (w/b) ratio are shown in Figure 5. The acetate-activated binders exhibited slightly higher fluidity than the hydroxide-activated binders, independently of the w/b ratio. At a w/b ratio of 0.3, the hydroxide slag pastes had no fluidity, whereas the acetate slag pastes were workable. Across both the hydroxide- and acetate-based systems, the K-based activators yielded a slightly higher fluidity than the respective Na-based activators. This is consistent with previous measurements on the relative fluidity of KOH-activated slag and NaOH-activated slag systems.^50^

**Figure 6:**
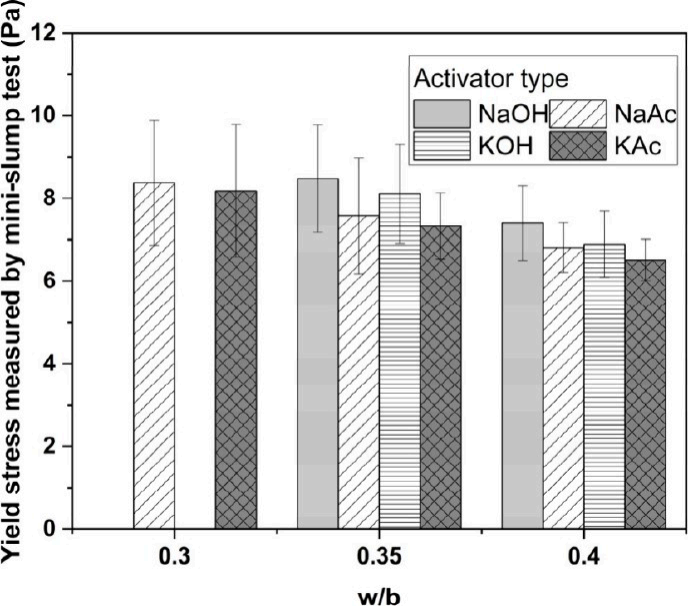
Yield stress calculated from mini-slump test results of activated slag binders as a function of the w/b ratio. Error bars represent one standard deviation of three measurements.

Figure 6 displays the calculated yield stress values of the assessed activated slag pastes, calculated using eq 1. Yield stress values of the hydroxide-activated binders are consistent with those reported by Tan et al.^35^ for other alkali-activated cements, produced with the same activator dosage of 4 wt % M_2_O used in this study and a w/b ratio of 0.4. The acetate-activated pastes exhibited a slightly lower yield stress compared with the hydroxide-activated pastes at w/b ratios of 0.35 and 0.4.

### 3.3 Phase Assemblage Evolution

**Figure 7:**
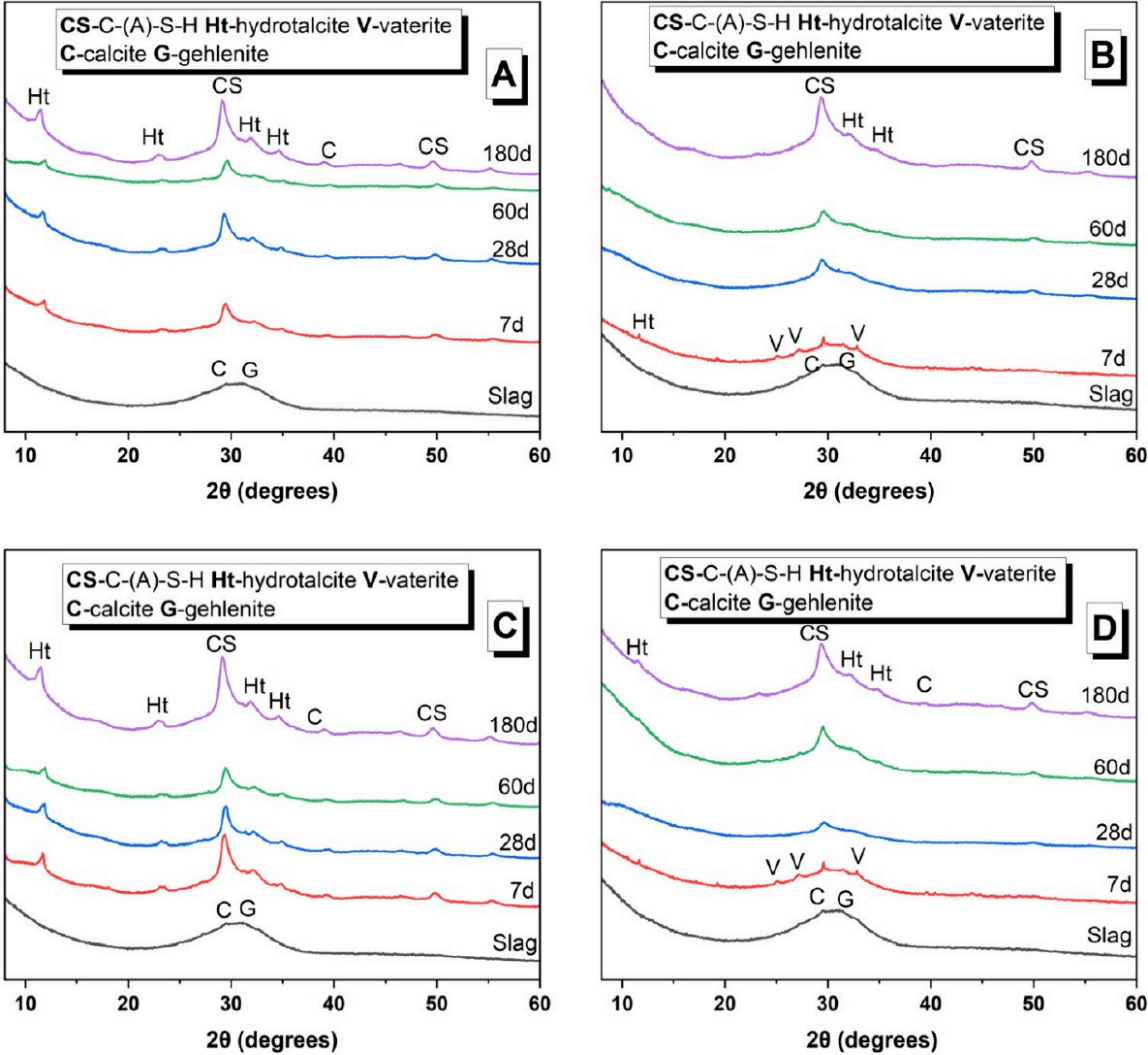
Cu Kα XRD patterns of AAS produced with different activators of (A) NaOH, (B) NaAc, (C) KOH, and (D) KAc curing at various days (relative to the mass of mixed paste).

The X-ray diffraction patterns of the binders produced with the hydroxide-based and acetate-based activators are shown in Figure 7A–D. Gehlenite (PDF #00-029-0285) was identified as the only crystalline phase in the unreacted slag. In the NaOH-based system (Figure 7A), two crystalline reaction products were identified: a calcium silicate hydrate (PDF #029-0329) and a layered double hydroxide with a hydrotalcite-type structure (PDF #01-089-0460), independently of the curing age, consistent with previous studies.^1^ In the KOH-AAS system (Figure 7C), the main crystalline reaction products were also a C-(A)-S-H-type gel and hydrotalcite at all curing ages. In both hydroxide-based binders, the main peak assigned to the C-(A)-S-H-type gels (2θ = 29.5°) became sharper and more intense at extended curing durations, consistent with an ongoing slag reaction.^51^

In the NaAc- and KAc-activated binders, the main reaction product observed was a C-(A)-S-H-type gel (Figure 7B,D). In both acetate-activated systems, the main peak assigned to the C-(A)-S-H-type gels (2θ = 29.5°) was visible only from 28 days of curing onward. For the NaAc-activated paste, a very small amount of C-(A)-S-H is expected to have formed by 7 days of curing (i.e., 168 h) since the maximum of the main heat flow peak occurred at 9.5 days (i.e., 228 h) (Figure 3A); it is therefore unsurprising that no C-(A)-S-H peak is visible in its 7 day XRD pattern (Figure 7B). For the KAc-activated paste, a higher intensity C-(A)-S-H is observed at 7 days (i.e., 168 h), consistent with the maximum of the heat flow peak occurring at ∼5.3 days (i.e., 128 h) (Figure 3B). However, the cumulative heat released for both the acetate-activated pastes by 168 h was significantly lower than that of the hydroxide-activated pastes (Figure 4). These observations indicate that the quantity of reaction products formed by 7 days in the acetate-activated pastes was sufficient to result in final setting (Table 4) but was too low to result in the formation of crystalline C-(A)-S-H (Figure 7B,D); but by 28 days, when the cumulative heat values of the acetate-activated pastes were double than those at 7 days, semicrystalline C-(A)-S-H is detectable in the XRD patterns.

The main basal reflection (003) of a hydrotalcite-type phase (Ht) (at 2θ ∼ 11.5°) was observed at 180 days of curing in both NaAc- or KAc-based binders, albeit with very weak intensity. Layered double hydroxides can be poorly crystalline in cementitious systems depending on their interlayer anion.^52^ It is therefore plausible that hydrotalcite formed at curing times earlier than 180 days but was not detectable by XRD. Minor traces of vaterite (PDF #01-074-1867) were identified after 7 days of curing for both NaAc and KOH binders. The occurrence of vaterite is most likely due to carbonation during sample handling,^53^ as this phase was only observed in 7 day cured samples, and it does not seem to be directly linked to the type of activator used.

In the NaAc-activated (Figure 7B) and KAc-activated (Figure 7D) binders, no reflections corresponding to crystalline sodium acetate or potassium acetate or their hydrates (Figure 1) were observed in the diffractograms: this indicates that these compounds had not precipitated during the curing process. Despite the slower reaction kinetics of acetate-activated binders compared with those of the hydroxide ones, similar crystalline reaction products were identified after 180 days of curing.

**Figure 8:**
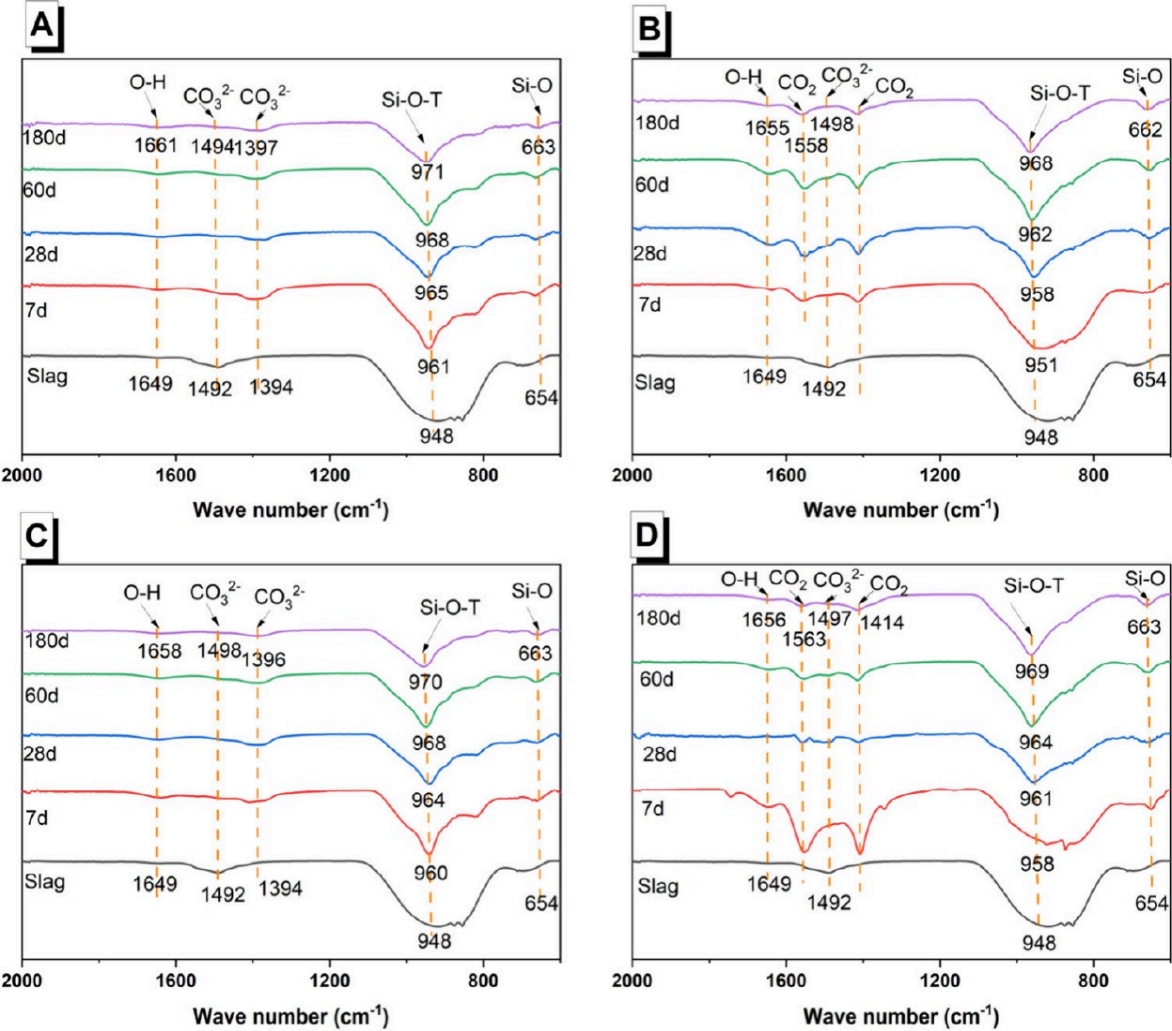
FTIR spectra of AAS produced with different activators of (A) NaOH, (B) NaAc, (C) KOH, and (D) KAc curing up to 180 days as a function of the curing age (relative to the mass of mixed paste).

FTIR spectra were collected for curing times up to 180 days (Figure 8A–D). These spectra can give information about the formation and structural evolution of the C-(A)-S-H-type gels formed and whether acetate groups were retained in the system. Si–O–T asymmetric stretching bands in the range of 951–971 cm^–1^ were observed, with a distinctive line shape that was narrower than the broad band from 800 to 1100 cm^–1^ in the slag, and were attributed to C-(A)-S-H-type gels.^54^ These narrow Si–O–T bands associated with C-(A)-S-H-type gels were clearly observed in the hydroxide-activated pastes’ spectra at all ages (Figure 8A,C), but in the acetate-activated pastes, they were only detectable at 28 days and beyond (Figure 8B,D). The absence of detectable absorption bands associated with C-(A)-S-H-type gels at 7 days is consistent with the similar observations on their XRD patterns (Figure 7B,D); these data support the interpretation that sufficient C-(A)-S-H was formed to achieve a final set before 7 days in the acetate-activated pastes, but the amount of C-(A)-S-H was still relatively small (in relation to the hydroxide-activated pastes).

A progressive shift in the asymmetric Si–O–T band maximum to higher wavenumbers with increasing curing times was observed for all systems. This phenomenon is attributed to an increasing degree of polymerization in the C-(A)-S-H-type gel over time.^55^ The deformation vibrations of Si–O bonds,^56^ located approximately at 663–662 cm^–1^, exhibited no significant differences between hydroxide-activated slag cements and acetate-activated slag cements. The FTIR spectra indicate that the local bonding environments within the C-(A)-S-H-type gels are very similar at later aging times (i.e., 28 days and onward) when using hydroxide- or acetate-based activators.

In hydroxide-activated binders, two bands associated with stretching vibrations ν[CO_3_]^2–^ were identified, one in the range of 1492–1498 cm^–1^ and the other in the range of 1394–1397 cm^–1^. These bands are typically attributed to carbonates;^57^ the calcite present in the anhydrous slag is expected to exhibit stretching vibrations ν[CO_3_]^2–^ in this wavenumber range.^58^ In the acetate-activated slag binders, bands were also present in the region of 1350–1500 cm^–1^, albeit at different positions to the bands observed in the hydroxide-activated samples: one band at 1418–1420 cm^–1^ and another at 1558–1563 cm^–1^. These two bands were respectively assigned to symmetric and antisymmetric stretching vibrations ν[CO_2_] and attributed to acetate group anions.^59^ Given that no characteristic peaks corresponding to unreacted sodium or potassium acetates were observed in the XRD patterns (Figure 7), it is possible that acetate groups have adsorbed onto surface sites of the reaction products.

**Figure 9:**
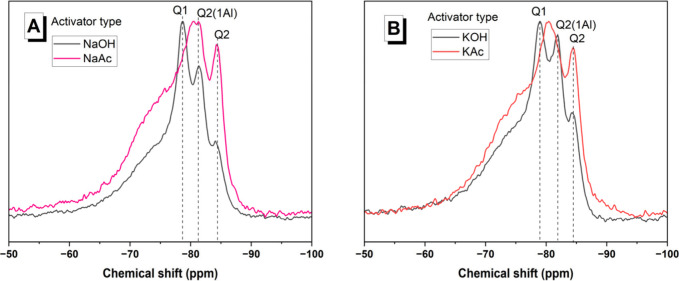
^29^Si MAS NMR spectra of 180 day activated slag binders produced with (A) Na-based or (B) K-based activators.

The structure and local chemical environments in the C-(A)-S-H-type gel were expected to be similar for both the hydroxide-activated and acetate-activated pastes at later aging times, from the evidence of the XRD patterns (Figure 7) and FTIR spectra (Figure 8). To investigate this further, ^29^Si MAS NMR spectra were collected for the slag-based pastes after 180 days of curing (Figure 9). The spectra of NaOH- (Figure 9A) and KOH-activated (Figure 9B) pastes were broadly similar; three separate resonances could be clearly identified and were assigned to Q^1^ (δ_obs_ = −79 ppm), Q^2^(1Al) (δ_obs_ = −82 ppm), and Q^2^ (δ_obs_ = −84 ppm), consistent with a C-(A)-S-H-type gel.^60^ The shoulder between δ_obs_ = −50 and −75 ppm corresponds to the unreacted slag.^43^

In both of the acetate-activated binders, only two peaks were clearly resolved. A Q^2^ resonance had very similar chemical shift values to those of the respective hydroxide-activated pastes’ spectra. A broader peak with a center at approximately δ_obs_ = −81 ppm was situated between the Q^1^ and Q^2^(1Al) resonance positions in the respective hydroxide-activated pastes’ spectra. This difference in spectral line shape between the hydroxide-activated and acetate-activated slags in this study is similar to previously reported spectral differences between sodium hydroxide-activated slags and sodium silicate-activated slags.^61^ Previous studies have interpreted this seeming single peak in alkali-activated slags’ spectra as being composed of separate, partly overlapping Q^1^ and Q^2^(1Al) resonances.^61−63^ Given that the activator anion type is known to affect the structure of binder gels in alkali-activated slag cements,^43^ it is plausible that the presence of acetate groups resulted in slight changes in the ordering of the silicate chains of the C-(A)-S-H-type gels forming, compared to those formed in hydroxide-activated slag.

### 3.4 Microstructure Features

**Figure 10:**
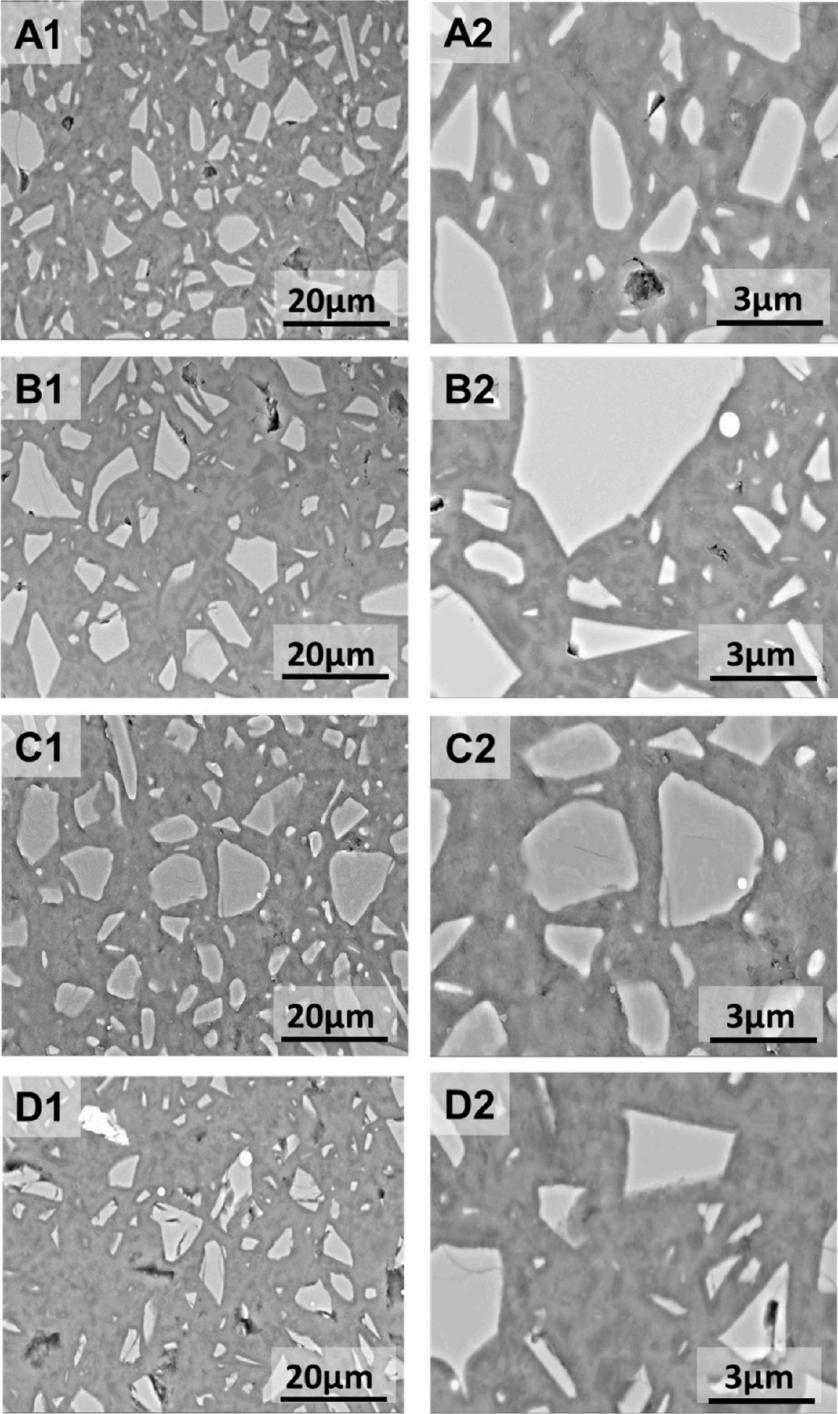
BSE images of the evaluated AAS cements after 180 days of curing at different magnifications: (A) NaOH-AAS, (B) NaAc-AAS, (C) KOH-AAS, and (D) KAc-AAS.

Figure 10 displays BSE images at two different magnifications of the activated binders after 180 days of curing. Unreacted slag particles are identifiable by their light gray color. Meanwhile, the medium gray areas surrounding correspond to binding phases, primarily comprising C-(A)-S-H-type gels along with secondary reaction products of hydrotalcite-like phases and carbonates, as identified from the XRD patterns (Figure 7).

No major differences were identified between samples; a homogeneous and dense continuous matrix was observed in all cases, independently of the type of activator used. A comparable microstructure in the NaOH- and KOH-activated binders is expected.^64,65^ Comparing the acetate-activated binders to the hydroxide-activated binders, the cumulative heat curves (Figure 4) and setting times (Table 4) indicated a much lower degree of slag reaction in the acetate-activated pastes at all curing times, consistent with the lower pH of the activating solutions (Figure 2); therefore, a less dense microstructure was therefore expected. However, neither large pores nor a disconnected microstructure was identified after 180 days; this can be explained by the ongoing reaction of slag at extended curing durations, as shown by the positive gradients of the alkali-activated pastes’ cumulative heat curves beyond 28 days (Figure 4), which were in fact higher than those of the hydroxide-activated pastes.

**Figure 11:**
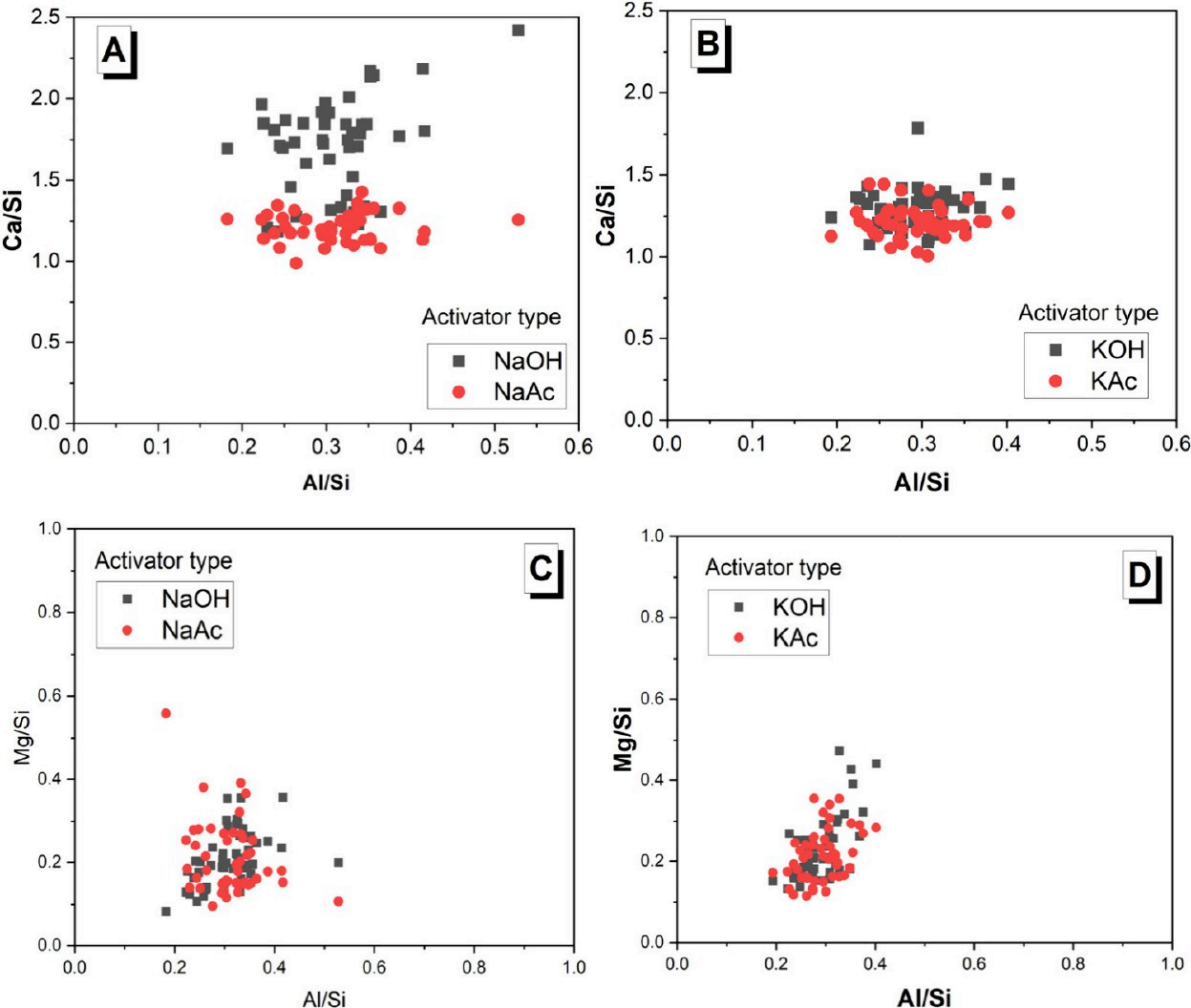
EDS results for 180 day cured (A, C) Na-based and (B, D) K-based binders. Results correspond to 47 measurements conducted in the main binding phase.

Results of the EDS analysis are listed in Figure 11. The Ca/Si ratios in the Na-based activated binders varied significantly when using NaOH or NaAc activators, being lower and more homogeneous in the case of using NaAc (Figure 11A). A comparable range of Al/Si ratios was detected for both activator types. This confirms that Al substitution is happening in the C-(A)-S-H-type gel forming in both systems, consistent with the observations from the ^29^Si MAS NMR results (Figure 9). In K-based activated binders, no significant differences in either Ca/Si or Al/Si ratio ranges were observed when using hydroxide- or acetate-type activators. The Al/Si ratios were below 0.8 across all pastes, in line with what has been previously reported for C-(A)-S-H.^66^ The average Ca/Si and Al/Si ratios are reported in Table 5.

**Table 5:** Ca/Si and Al/Si Atomic Ratios of 180 Day Cured Activated Slag Binders as a Function of the Activator Type^a^

activator type	Ca/Si ratio	Al/Si ratio
NaOH	1.70 ± 0.30	0.31 ± 0.06
NaAc	1.21 ± 0.09	0.29 ± 0.05
KOH	1.30 ± 0.12	0.29 ± 0.04
KAc	1.21 ± 0.10	0.28 ± 0.05

a Values correspond to the average and one standard deviation of 47 measurements conducted in the main binding phase.

Figure 11C,D shows the correlation between Mg/Si and Al/Si, providing robust evidence of the formation of a Mg–Al layered double hydroxide (LDH) with a hydrotalcite-like structure, as identified in other alkali-activated slag binders^67^ and consistent with the XRD results (Figure 7). A more pronounced scattering of the Mg/Al ratio was observed in the Na-based activated slag binders, while the Mg/Al ratios of the K-based activated binders were comparable. The XRD patterns for 180 day cured samples clearly showed the formation of a hydrotalcite-type phase in the hydroxide-activated slag binders (Figure 7A,C), whereas much weaker peaks were attributed to a hydrotalcite-type phase in the acetate-activated pastes’ patterns at 180 days (Figure 7 B,D). Collectively, these results suggest that the formation of a Mg–Al LDH is still happening in the presence of an acetate-type activator but with a poorly crystalline structure.

**Figure 12:**
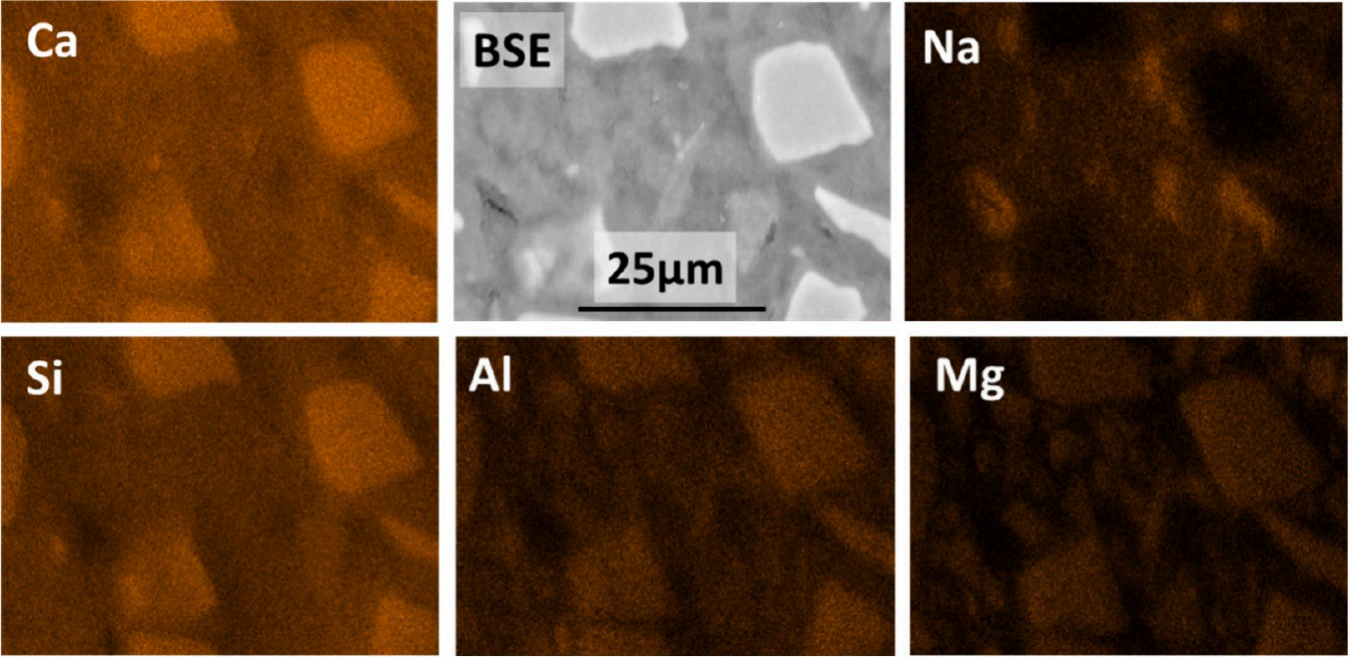
BSE and EDS elemental maps of 180 day cured NaOH-activated slag paste.

Elemental map analysis of NaOH-based slag binders is shown in Figure 12, where enriched Ca regions, consistent with the unreacted slag particles, and a homogeneous distribution of Ca throughout the binding phase, consistent with the formation of a C-(A)-S-H-type gel, can be observed. Al, Si, and Mg are also enriched in the unreacted slag particles, and the Al and Si distributions in the samples seem to follow a similar trend to that observed in the Ca maps. Of interest, discrete Na-rich regions consistent with fully reacted slag particles were identified. In those regions, Al or Mg were not clearly identified, which might suggest that Na is chemically bonded to a Ca- and Si-rich phase, consistent with a C-(N)-A-S-H-type gel typically identified in alkali-activated slag cements.^68^

**Figure 13:**
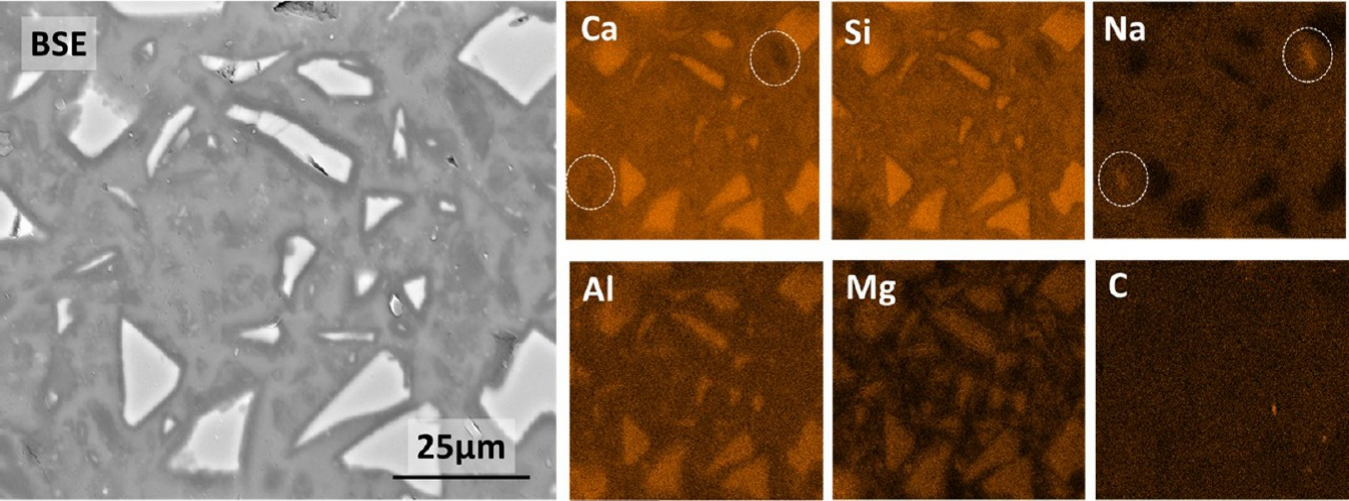
BSE and EDS elemental maps of 180 day cured NaAc-activated slag paste. The Na-rich particle is circled in the Na map, showing potential formation of N-A-S-H.

Similar to the NaOH-activated binders, when using a NaAc activator (Figure 13), Ca-, Si-, Al-, and Mg-rich regions corresponding to the unreacted slag particles are observed. In this case, a distinctive rim around some of the large slag particles (dark gray) are observed, which are rich in Mg and Al, consistent with the formation of hydrotalcite phases, as observed in other NaAc-activated slag systems.^69^ In this case, a more homogeneous distribution of Na is observed in the binding phase; however, a discrete Na-rich particle was observed where no Ca, Si, or Mg were present. The C map does not show any enriched intensity in the region where the Na-rich particle was observed; therefore, it is unlikely that this corresponds to unreacted NaAc. For alkali-activated slag cements, it has been hypothesized that depending on the activator type (particularly when using sodium silicate), the formation of a N-A-S-H-type gel is possible at extended curing ages.^43^ The results suggest that when using an acetate-type activator at advanced curing ages, preferential formation of this phase might be happening.

**Figure 14:**
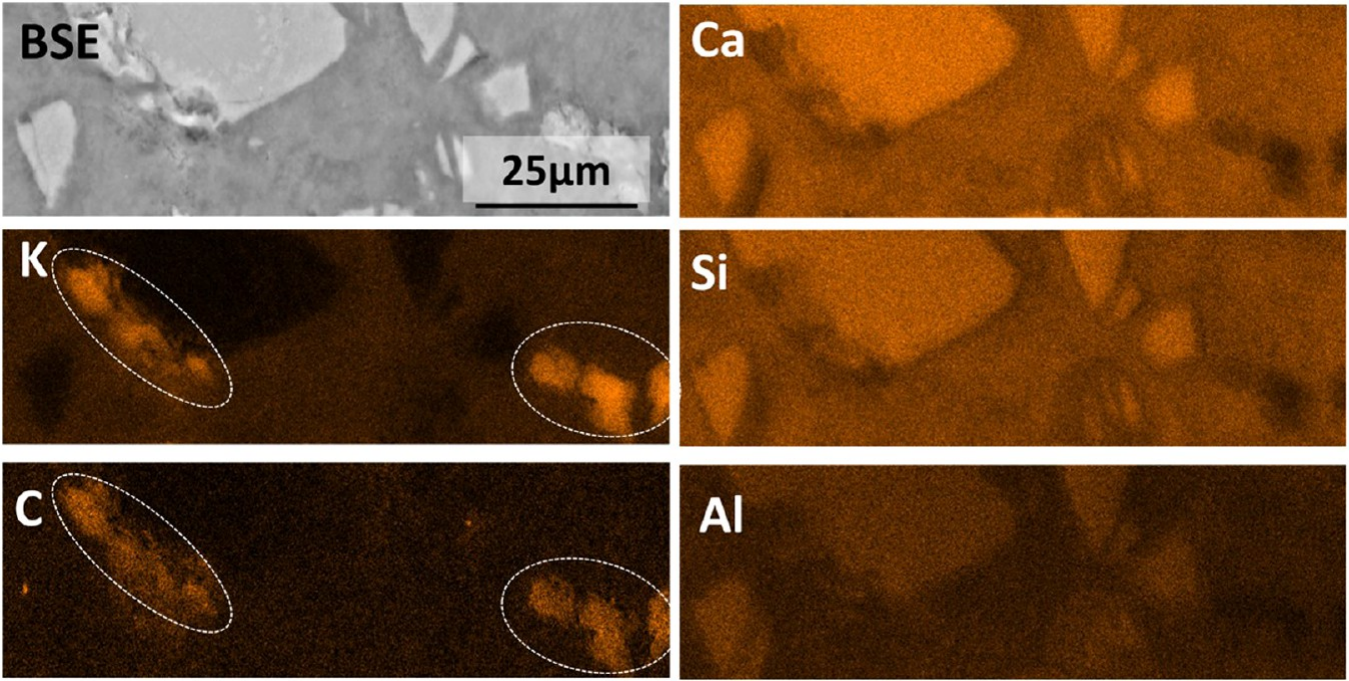
BSE and EDS elemental maps of 180 day cured KAc-activated slag paste. K-rich and C-rich regions are circled in the K and C maps to show precipitation of amorphous KAc.

The elemental maps for a KAc-based binder are shown in Figure 14, where regions rich in K and C can be clearly seen around unreacted slag particles without Ca, Si, Al, or Mg. This suggests the precipitation of KAc. However, no reflections of crystalline KAc were identified by XRD (Figure 7D) in these samples, suggesting that any KAc precipitating on the surface of the slag particles is poorly crystalline.

### 3.5 Compressive Strength and Porosity

**Figure 15:**
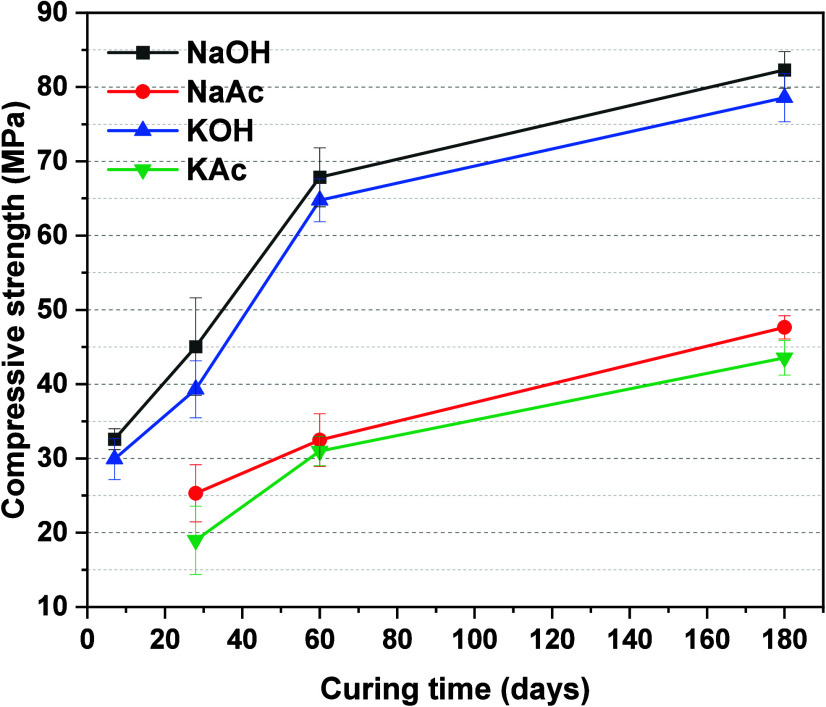
Compressive strength of slag-based pastes as a function of activator type and curing age.

Figure 15 shows the compressive strength evolution of paste cubes up to 180 days of curing. In the case of the acetate-activated binders, it was not possible to record compressive strength values before 7 days of curing, as the samples did not harden before this age, consistent with the delayed reaction identified by isothermal calorimetry (Figure 3) and setting times (Table 4). After 28 days of curing, all samples had hardened. The 28 day strengths of the samples activated with NaAc (25.3 MPa) and KAc (19.0 MPa) were lower than those of the samples activated using NaOH (45.1 MPa) and KOH (39.3 MPa). Considering the comparable microstructures of the acetate-activated pastes compared to the hydroxide-activated pastes at 180 days (Figure 10), it might be expected that the compressive strengths of all pastes would be similar at 180 days. However, at all curing durations, the strengths of the acetate-activated pastes were significantly lower than those of the hydroxide-activated materials. This suggests that other factors caused the lower compressive strength of the acetate-activated pastes. A steady strength gain was observed in the acetate-activated pastes from 28 to 180 days. This indicates that the reaction of the slag in acetate-activated systems continued beyond 28 days, as suggested by the cumulative heat curves (Figure 4). The compressive strength values achieved in the acetate-activated pastes are sufficiently high for producing grouts, blocks, or paver.

**Figure 16:**
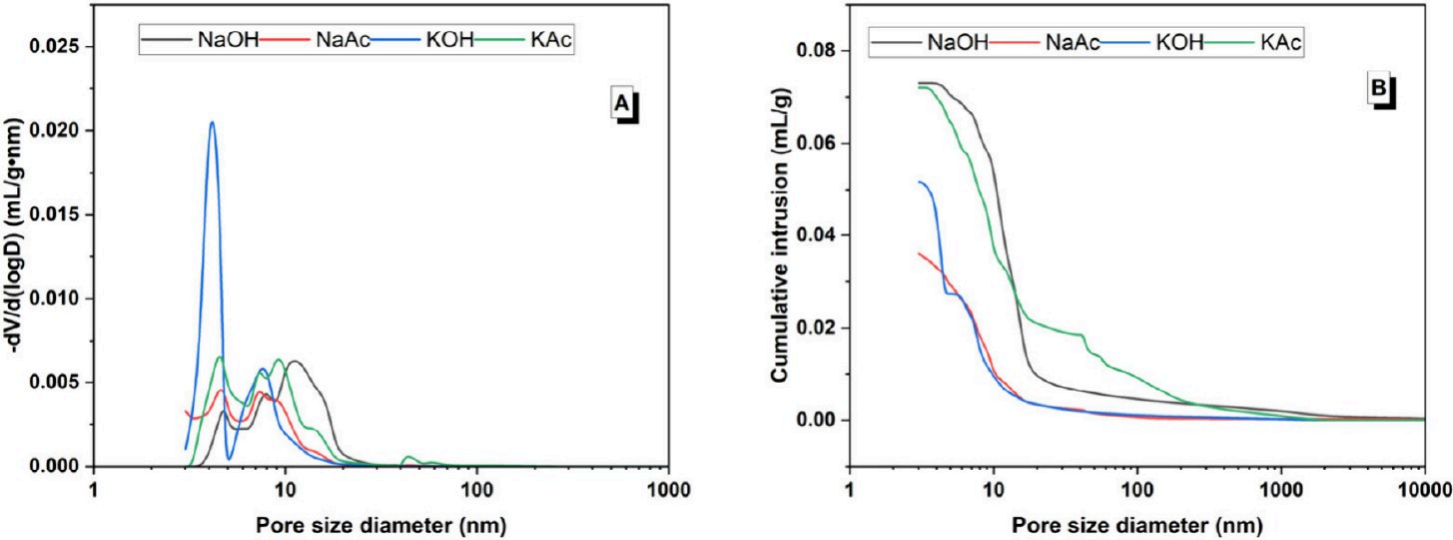
Pore size distribution of alkali-activated slag pastes at 28 days as a function of different activators. (A) Differential pore volume and (B) cumulative pore volume.

Compressive strength development is not only influenced by the phase assemblages of a cementitious matrix; it can also be affected by the pore structure. Figure 16A,B shows mercury intrusion porosimetry results for the 28 day cured AAS pastes. Unlike the 28 day strength results, there was not a clear and consistent difference between acetate-activated and hydroxide-activated pastes: the NaAc-activated binder exhibited a lower cumulative intrusion volume compared to the NaOH-activated slag, while the KAc-activated binder exhibited a higher cumulative porosity volume compared to the KOH-activated slag (Figure 16B). Previous research on hybrid organic–inorganic alkali-activated slag cements found that the presence of an organic phase in the cured samples’ microstructure reduced the total porosity and shifted the pore distribution to smaller sizes;^23^ such an effect was not seen in these acetate-activated systems.

**Figure 17:**
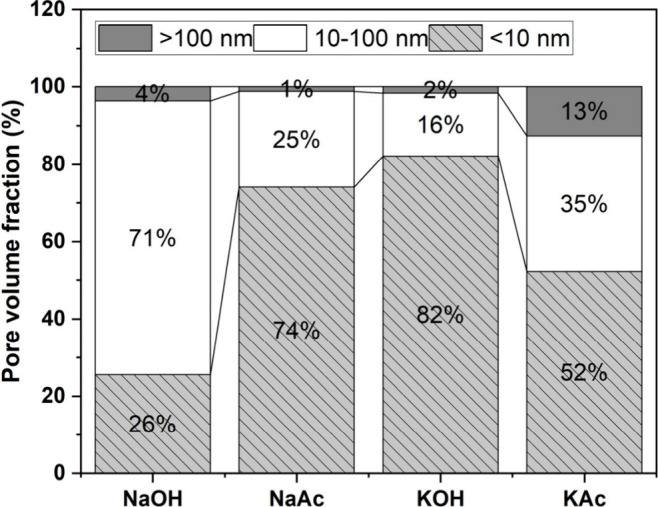
Pore size fraction of 28 day cured alkali-activated slag binders as a function of the activator type.

The porosity distributions were classified into size categories of <10 nm, 10–100 nm (i.e., micropore range), and >100 nm (i.e., macropore range)^70^ (Figure 17), and the critical pore entry size diameter and total porosity values for each sample are listed in Table 6. The >100 nm pore size range made up the smallest proportion of pores (≤13 vol %) across all samples, whereas there was much more variation for the 10–100 nm (16–71 vol %) and <100 nm (26–82 vol %) pore size ranges (Figure 17). The proportion of macropores (i.e., >100 nm) was highest for the pastes activated with NaOH (4 vol %) and KAc (13 vol %); these pastes also had the highest total porosity (14.0 and 14.8 vol %, respectively) (Table 6). The critical pore size corresponds to the maximum value observed in the differential distribution curves, as well as the pore size occurring in the highest frequency in the interconnected pores.^71^ A comparable critical pore size of 9–10 nm is identified in all the activated binding pastes except for the KOH-activated paste, which had a lower critical pore size of 4 nm (Table 6). However, ultimately, there were no clear and consistent differences between the porosity characteristics of the acetate-activated and hydroxide-activated pastes. There does not seem to be a clear correlation between the pore structure of the binders produced and the compressive strength developed after 28 days of curing.

**Table 6:** Critical Pore Diameter and Total Porosity of 28 Day Activated Slag Cement Pastes

paste type	critical pore size diameter (nm)	total porosity (vol %)
NaOH-AAS	10	14.0
NaAc-AAS	9	8.5
KOH-AAS	4	10.9
KAc-AAS	9	14.8

Further studies are required to determine the degree of slag reaction, the nanostructure features of the reaction products forming, and the pore structure determined via nondestructive techniques in order to elucidate the factors controlling the acetate-activated slag binders’ performance.

### 3.6 Wettability of Activated Slag Pastes

**Figure 18:**
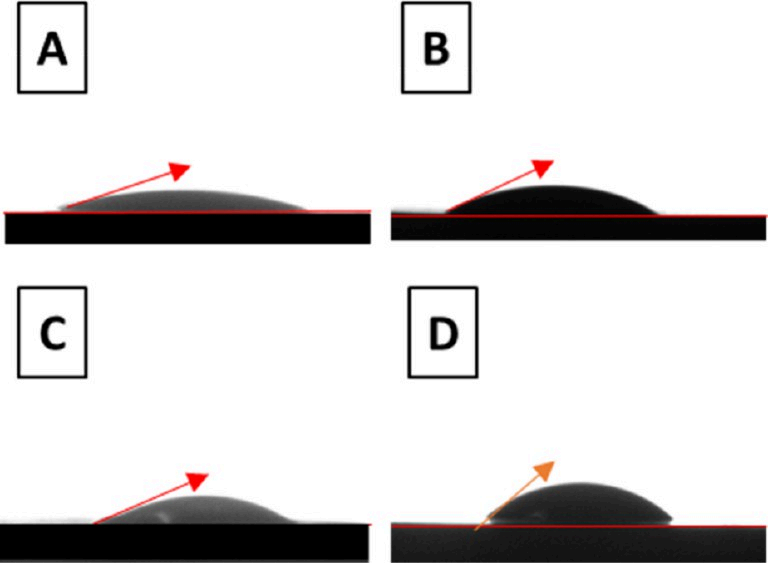
Photographs showing contact angle in 180 day cured slag cements produced with (A) NaOH-, (B) NaAc-, (C) KOH-, and (D) KAc-based activators.

Water contact angle measurements give an indication of affinity between a solid surface and surface water: a smaller contact angle represents better surface wettability.^72^Figure 18 shows photographs showing the water contact angle in different 180 day alkali-activated binders, with the values of the contact angles reported in Table 7. After 28 days of curing, the water contact angle could not be obtained for the NaOH- or KOH-activated pastes, as the water completely covered the surface, indicating perfect wetting and/or water absorption of these materials. Conversely, for the acetate-activated binder, a clear water contact angle was observed at 28 days, indicating less hydrophilicity than the hydroxide-activated pastes. At 180 days of curing, a water contact angle could be obtained for all of the evaluated activated slag cements. NaAc-activated pastes exhibited a slightly higher contact angle than NaOH-activated pastes, indicating that using NaAc leads to a slightly lower hydrophilicity. Similarly, using a KAc activator leads to a slightly higher contact angle than when using KOH.

**Table 7:** Average Contact Angle of Activated Slag Cement Pastes as a Function of Activator Type and Curing Age^a^

	curing age (days)	
activator type	28	180
NaOH	-	34.33° ± 0.14°
NaAc	44.21° ± 0.01°	43.09° ± 0.11°
KOH	-	42.17° ± 0.01°
KAc	49.95° ± 0.07°	46.12° ± 0.02°

a Error corresponds to one standard deviation of two measurements.

This approach for attempting to enhance hydrophobicity can be classified as “integral mixing”, as the hydrophobic agent is mixed within the cement paste, rather than applying a surface-based treatment only.^73^ A two-way mechanism for the enhanced impermeability from sodium acetate addition was proposed in previous studies: first, precipitation of sodium acetate in pores helped to densify the microstructure; second, the bonding of organic functional groups with silicon atoms in the C-S-H gel gave a greater hydrophobic character to the microstructure.^74,75^ However, for the activated slag samples in this study, the densification part of the previously proposed mechanism is not applicable. As shown in Table 6, the acetate-activated binders present the lowest and highest total porosities of the four samples evaluated at 28 days. Yet, the acetate-activated pastes exhibited less hydrophilicity at 28 days, whereas the hydroxide-activated pastes were sufficiently hydrophilic that a contact angle could not be successfully measured. The second part of the previously proposed mechanism around hydrophobic functional groups^76^ is more plausible for these systems. The activator anion type is the only variable that matches the trends observed for the contact angle values. It is plausible that increased hydrophobicity can be achieved through surface functional groups in alkali-activated systems as well as Portland cement-based systems (which have been the focus of most previous studies). The addition of sodium methyl silicate to a sodium hydroxide- and sodium silicate-activated metakaolin geopolymer achieved highly hydrophobic behavior; this was attributed to the presence of alkyl surface groups on the N-A-S-H gel.^13^ The FTIR spectra (Figure 8) suggest that it is likely that acetate groups are still present in the acetate-activated slag at 28 days of curing, and the BSE-EDS results for the KAc-activated paste at 180 days (Figure 14) support this interpretation. Changes in the hydrophilicity of the binder would need to occur at the solid–liquid interface. Given that acetate anions are not consumed (or at least not fully consumed) in the formation of C-(A)-S-H-type gels, it is plausible that interactions between the C-(A)-S-H-type gel and acetate anions in pore solution may be occurring during the curing process.

Therefore, the decreased wettability in these acetate-activated slag pastes was attributed solely to the hydrophobic effect of acetate functional groups with no consistent pore densification effects observed. This behavior is distinct from previous studies using alkali acetate as an impermeability enhancing agent, in which increased hydrophobicity is attributed to both pore densification and the hydrophobic effect of acetate functional groups.^74,75^ This difference is attributed to the fact that in this study, sodium acetate is used as the activator and is partly consumed in the formation of binder phases, whereas in the conventional use of sodium acetate as an additive, it is not consumed in product phase formation and instead precipitates out of solution within the pores.^74,75^

### 3.7 Proposed Reaction Mechanism of Alkali Acetate-Activated Slag Cements

**Figure 19:**
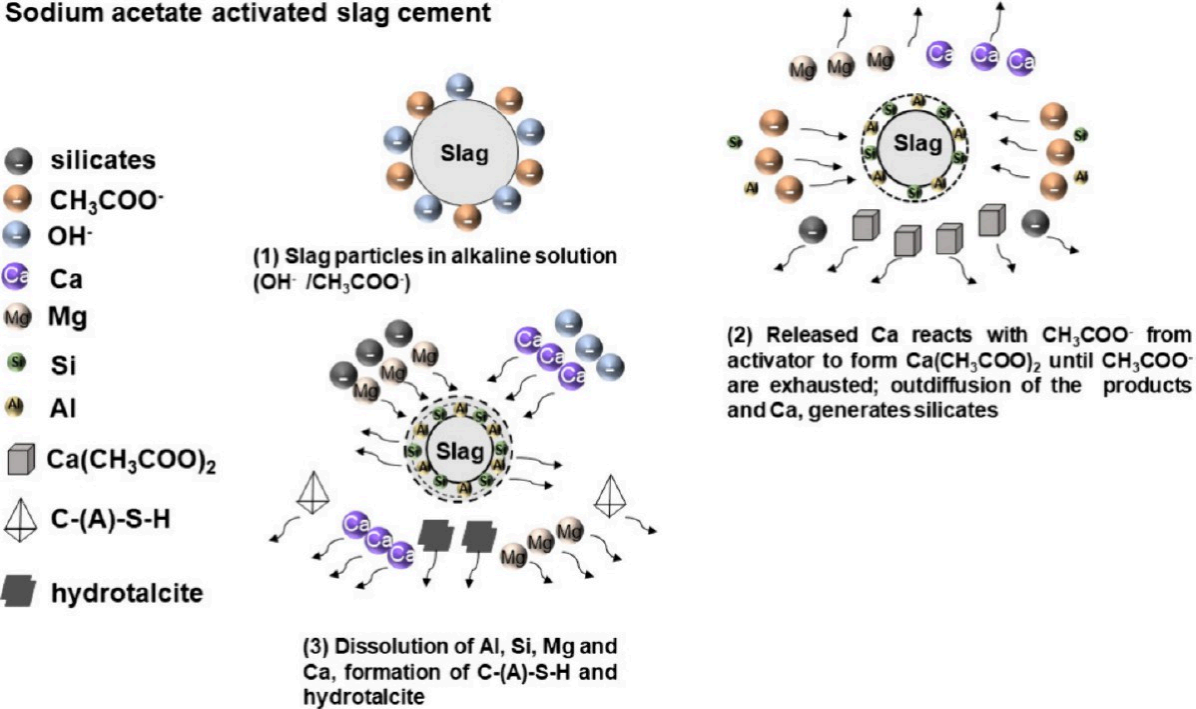
Schematic diagram illustrating the proposed reaction mechanism of alkali acetate-activated slag pastes.

Figure 19 illustrates the proposed reaction process in alkali metal acetate-activated slag cements. The dissociation of NaAc in solution, as represented in eqs 2 and 3, produces OH^–^ ions that aid the dissolution of different species from the slag as the reaction progresses. The Ca^2+^ ions released from the dissolved slag are likely to react with the CH_3_COO^–^ ions dissociated from NaAc in the solution, resulting in the formation of calcium acetate, along with sodium silicate, as depicted in eq 4 below. The formation of aqueous calcium acetate complexes has previously been found to occur when potassium acetate is used as a deicing agent in Portland cement pastes, in which Ca^2+^ cations from portlandite dissolution form complexes with acetate anions in solution.^29^
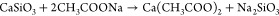
4

The formation of sodium silicate, which is a conventional activator used for producing alkali-activated cements, will favor the continued dissolution of slag, and a competitive reaction toward the formation of calcium acetate via a complexation with precipitation of C-(A)-S-H-type gels is likely to take place. The C-(A)-S-H-type gels formed in the acetate-activated pastes indeed exhibited similarities with those formed in sodium silicate-activated slags, in terms of the ^29^Si MAS NMR spectral line shape^61^ (Figure 9) and the slightly lower Ca/Si ratio compared to those of the hydroxide-activated slags^53,77^ (Figure 11).

At early times of reaction, it is also likely that any C-(A)-S-H precipitating reacts with the sodium acetate in solution (as per eq 4), consequently driving the preferential formation of calcium acetate until the CH_3_COO^–^ ions are depleted. This phenomenon might explain the longer induction period observed in the calorimetry results of NaAc/KAc-AAS. A substantial delay before the occurrence of maximum heat flow of the main hydration peak has also been observed when using weak sodium silicate activators,^78^ albeit less extreme than the durations seen here (Figure 3). There are similarities with the proposed mechanism for sodium oxalate (Na_2_C_2_O_4_) activation of basic oxygen furnace steel slag, in which dissociated oxalate anions in solution bond with Ca^2+^ cations leached from the slag to form calcium oxalate.^79^

Calcium acetate is highly hygroscopic and readily forms hydrates. From a previous study on calcium acetate hydrate and calcium acetate hemihydrate,^80^ their most intense XRD peak is at ∼9° (2θ), thermal decomposition occurs at ∼390 °C, and they exhibit two groups of overlapping resonances in their FTIR spectra between 1350 and 1600 °C. None of these key features were identified in the acetate-activated binders produced here. Hence, it is plausible that acetate groups are bound to the C-(A)-S-H and/or Mg–Al LDH, which could explain the structural changes and compositions identified in these reaction products as a function of the activator type.

Testing this hypothesized reaction mechanism would require detailed investigation of the pore solution at different aging times, which could confirm whether calcium acetate and sodium silicate are forming as intermediary compounds in solution. The lower pH of the NaAc activating solution compared to that of the KAc activating solution (Figure 2) is a straightforward explanation for the slower reaction kinetics of the NaAc-activated slag pastes compared to those of the KAc-activated pastes (Figure 3, Table 4). However, the multistep nature of the proposed reaction mechanisms means it is necessary to understand various potential influences on solution speciation: for example, differences in solubility^32^ and dissociation constants^81^ between KAc and NaAc and the effect of buffering reactions induced by the dissolution of atmospheric carbon dioxide.^41^ Nonetheless, on the basis of the experimental evidence available so far, the proposed mechanism is the simplest and most plausible mechanism to explain the reaction kinetics and phase assemblage formation of alkali acetate-activated slags.

## 4 Conclusions

The feasibility of using NaAc and KAc to produce hybrid organic–inorganic slag-based binders was evaluated, and the properties of the resulting binders were determined. Acetate-activated slag cements exhibited a much longer induction period in the initial hours after mixing. Consequently, the initial setting times (up to 3 h) and final setting times (up to 230 h) were longer compared with those of NaOH- or KOH-activated slag binders.

The main reaction products identified in acetate-activated slag cements were C-(A)-S-H-type gels and layered double hydroxide (LDH)-type phases, with no significant differences in the types of reaction products between acetate- and hydroxide-based binders. However, XRD analysis showed variations in peak intensities, indicating differences in reaction kinetics and the time of hydrotalcite phase formation, which appeared much later in acetate-activated binders (180 days) compared to hydroxide-activated binders (7 days). ^29^Si MAS NMR spectroscopy suggested slight microstructure changes in C-(A)-S-H-type gels when using acetate activators compared with hydroxide-activated pastes, consistent with the slight chemical composition variations in C-(A)-S-H-type gels identified by BSE-EDS analysis. Significant differences were noted in the LDH with different Mg/Al ratios, especially with KAc activators. Further studies are needed to analyze the degree of Al substitution in C-(A)-S-H and the degree of slag reaction depending on the activator type to elucidate structural changes induced by acetate activators.

The compressive strength of hydroxide-activated binders was consistently higher than that of acetate-activated pastes at all curing ages, up to 180 days. Water contact angle measurements were higher for acetate-activated binders up to 49.95° ± 0.07°, indicating greater impermeability, likely due to the hydrophobic nature of acetate ions intermixed with reaction products.

These results suggest that alkali acetates can serve as effective activators for slag-based cements, promoting the development of microstructures and phase assemblages similar to those of hydroxide-activated cements. However, optimizing acetate-activated binders is necessary to enhance reaction kinetics and compressive strength, potentially by blending with conventional alkaline activators. The reduced hydrophilicity of alkali acetate-activated binders makes them attractive for applications requiring moderate strength and reduced permeability.

## Data Availability

The database of the results reported in this study is available at https://doi.org/10.5518/1548.
